# A Three-Zone Classification System for Biomaterial-Based Management of Deep Subgingival Carious Lesions

**DOI:** 10.3390/jcm15187209

**Published:** 2026-09-17

**Authors:** Masoud Hassan Zadeh, Carlos A. Jurado, Kiarash Karimi, Silvia Rojas-Rueda, Damian J. Lee, Hamad Algamaiah, Abdullah Almansour, Abdulrahman Alshabib

**Affiliations:** 1 Private Practice, 9203 ZX Drachten, The Netherlands; 2 School of Dental Medicine, Ponce Health Sciences University, Ponce 00716, Puerto Rico; 3 Department of Restorative Sciences, School of Dentistry, University of California, Los Angeles, CA 90024, USA; 4 Private Practice, Woodland Hills, Los Angeles, CA 91357, USA; 5 Department of Prosthodontics, Tufts University School of Dental Medicine, Boston, MA 02111, USA; 6 Department of Restorative Dental Science, College of Dentistry, King Saud University, Riyadh 11545, Saudi Arabia; 7 College of Dentistry, Prince Sattam Bin Abdulaziz University, Al-Kharj 11942, Saudi Arabia

**Keywords:** classification, composite resins, dental caries, dentistry

## Abstract

**Background:** This article introduces a clinically oriented, anatomy-based classification of subgingival margins. The proposed system categorizes margins into distinct zones based on their relationship with gingival and periodontal tissues and links each zone to appropriate restorative strategies. **Methods:** The classification is based on established principles of periodontal anatomy and restorative dentistry, as well as clinical considerations related to accessibility, isolation, and matrix placement. Three zones are proposed: (1) margins located within the free gingiva, which are generally accessible and manageable with rubber dam isolation; (2) margins extending into the junctional epithelium and supracrestal tissue attachment, where additional measures, including matrices and wedges, are often required to achieve adequate isolation and adaptation; and (3) margins located at or below the alveolar bone crest, which have traditionally required surgical management but may, in selected cases, be managed with advanced matrix techniques. **Results:** The proposed classification helps clinicians assess the complexity of subgingival margins before performing deep margin elevation (DME) and provides guidance for selecting appropriate clinical procedures according to margin depth and anatomical location. **Conclusions:** This classification provides a practical framework for selecting appropriate isolation techniques and treatment strategies to improve the predictability of restorative outcomes. Further clinical research is needed to evaluate its validity and its effects on periodontal health and long-term restorative outcomes.

## 1. Introduction

Subgingival dental lesions represent a significant clinical challenge because of their location, limited accessibility, and proximity to the periodontal tissues [1,2]. Deep carious lesions, cervical defects, fractures, and previously placed restorations may result in restorative margins located subgingivally, making visualization, isolation, caries removal, matrix placement, and adhesive procedures considerably more difficult. When these lesions are extensive and compromise the restorability of the tooth, extraction may ultimately be considered. Deep carious lesions remain an important cause of tooth loss, and once extraction is performed, clinicians must provide patients with appropriate replacement options, which may include conventional fixed dental prostheses, removable partial dentures, or implant-supported restorations [3,4].

However, these treatment alternatives are not always feasible or desirable. Certain patients may have systemic conditions, complex medical histories, or medications that increase the risks associated with dental extraction, periodontal surgery, or implant placement. In other situations, anatomical limitations, patient preferences, or financial constraints may prevent the use of implant-supported, tooth-supported, or removable prosthetic treatment [5,6,7]. Consequently, preservation of the natural tooth, whenever biologically and restoratively feasible, may represent an important treatment objective. In selected clinical situations, procedures that extend beyond conventional restorative approaches, such as deep margin elevation (DME), may provide an alternative for maintaining teeth that might otherwise be considered difficult to restore [8].

Deep margin elevation has emerged as a conservative restorative technique intended to relocate a deep subgingival cervical margin to a more accessible and coronal position. The procedure generally involves placement of a resin-based restorative material in the deep proximal or cervical portion of the cavity before completion of the definitive direct or indirect restoration. By elevating the margin, the clinician may improve visualization, facilitate rubber dam isolation, enhance matrix adaptation, simplify impression or digital scanning procedures, and provide better access for adhesive cementation and finishing. In appropriately selected cases, DME may also reduce the need for surgical crown lengthening and thereby preserve periodontal tissues and sound tooth structure [9,10,11].

Despite these potential advantages, the predictability of DME depends heavily on the location of the restorative margin relative to the surrounding periodontal structures [12]. A margin confined to the free gingival region presents a very different clinical scenario from one extending into the junctional epithelium, supracrestal tissue attachment, or to the level of the alveolar bone crest. As the margin becomes progressively deeper, the clinician encounters greater difficulties with visualization, moisture control, hemostasis, matrix placement, contour development, and removal of restorative excess. These factors may directly influence marginal adaptation, adhesive performance, emergence profile, and the long-term response of the periodontal tissues.

Management becomes particularly challenging when restorative margins approach or invade the tissues of the supracrestal attachment. In these areas, bleeding and crevicular fluid can compromise the adhesive field, while inadequate matrix adaptation may result in overhangs, deficient proximal contours, or residual restorative material within the gingival sulcus. Such deficiencies may favor plaque accumulation and contribute to periodontal inflammation. Furthermore, periodontal anatomy is not uniform. Variations in gingival thickness, sulcus depth, supracrestal tissue dimensions, bone morphology, tooth position, and interproximal architecture may exist between patients and even between different sites in the same patient. Therefore, the clinical significance of a subgingival margin cannot be determined solely by describing it as “deep” or “subgingival.”

Although the management of subgingival restorative margins and their biological implications have been extensively discussed, a clear and clinically applicable classification that integrates anatomical depth, accessibility, periodontal relationships, and restorative management remains lacking. Current terminology does not consistently differentiate margins that can be predictably isolated and restored from those requiring advanced matrix techniques, periodontal intervention, or alternative treatment strategies. Consequently, treatment planning may rely considerably on the clinician’s individual experience and subjective assessment.

A clinically oriented, anatomy-based classification of subgingival margins could provide a more standardized framework for diagnosis and treatment planning. By relating the position of the restorative margin to specific periodontal structures and to the degree of clinical accessibility, such a classification may help clinicians anticipate procedural difficulty, select appropriate isolation and matrix techniques, determine when DME is feasible, and recognize situations in which surgical or alternative treatment approaches should be considered. Establishing this framework may also facilitate communication among clinicians and investigators and provide a more consistent basis for evaluating the biological and restorative outcomes associated with DME.

## 2. Materials and Methods

This article was designed as a conceptual framework grounded in literature-based anatomical reasoning and clinical restorative principles. The proposed classification was developed by integrating evidence on the dentogingival unit, supracrestal tissue attachment, restorative margin placement, and deep margin elevation (DME). In particular, the framework was based on the recognized anatomical distinction between the gingival sulcus/free gingiva, the junctional epithelium plus supracrestal connective tissue attachment, and the relationship of these soft-tissue structures to the alveolar crest. Because the dimensions of the dentogingival junction are biologically variable between individuals and tooth sites, the classification was intentionally structured around clinically identifiable anatomical zones rather than fixed numerical cutoffs alone.

The clinical component of the classification was informed by operative observations routinely used in treatment planning for subgingival margins, including the estimated depth of the defect by periodontal probing and radiographic assessment, as well as the anticipated difficulty of access, matrix adaptation, and isolation during adhesive procedures. The rationale for linking anatomy to technique was drawn from literature showing that subgingival restorative margins are more difficult to manage biologically and technically, are associated with greater periodontal risk when they extend apically, and that DME has been proposed as a conservative method to relocate deep cervical margins coronally, although available evidence remains limited and partly conflicting. Accordingly, the present classification should be considered a hypothesis-generating clinical framework intended to support instrument selection and treatment planning, not a validated prognostic system.

### 2.1. Proposed Classification

**Figure 1:**
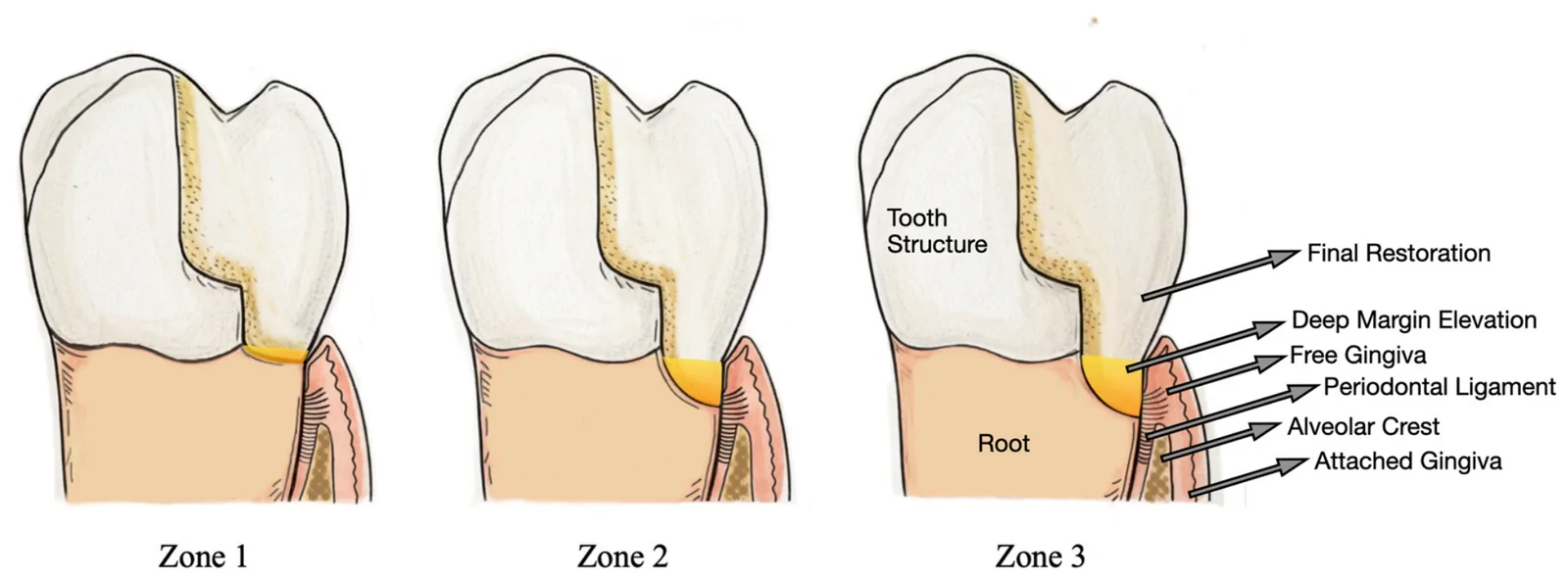
Zone classification. Zone 1: Margins within the free gingiva (supragingival/shallow subgingival); Zone 2: Margins within the junctional epithelium and supracrestal tissue attachment; Zone 3: Margins at or below the alveolar bone crest (intraosseous level).

The classification presented here divides subgingival margins into three anatomical zones, based on their relationship to the surrounding periodontal tissues and how manageable they are during restorative procedures. The goal is to offer a practical way of linking the biological position of a margin to its clinical handling, particularly in the context of deep margin elevation (DME). Because the dimensions of the supracrestal tissue attachment can vary between patients and sites, the boundaries between these zones should be seen as clinical guidelines rather than exact, measurable limits (Figure 1).

### 2.2. Zone 1—Margins Within the Free Gingiva (Supragingival/Shallow Subgingival)

**Figure 2:**
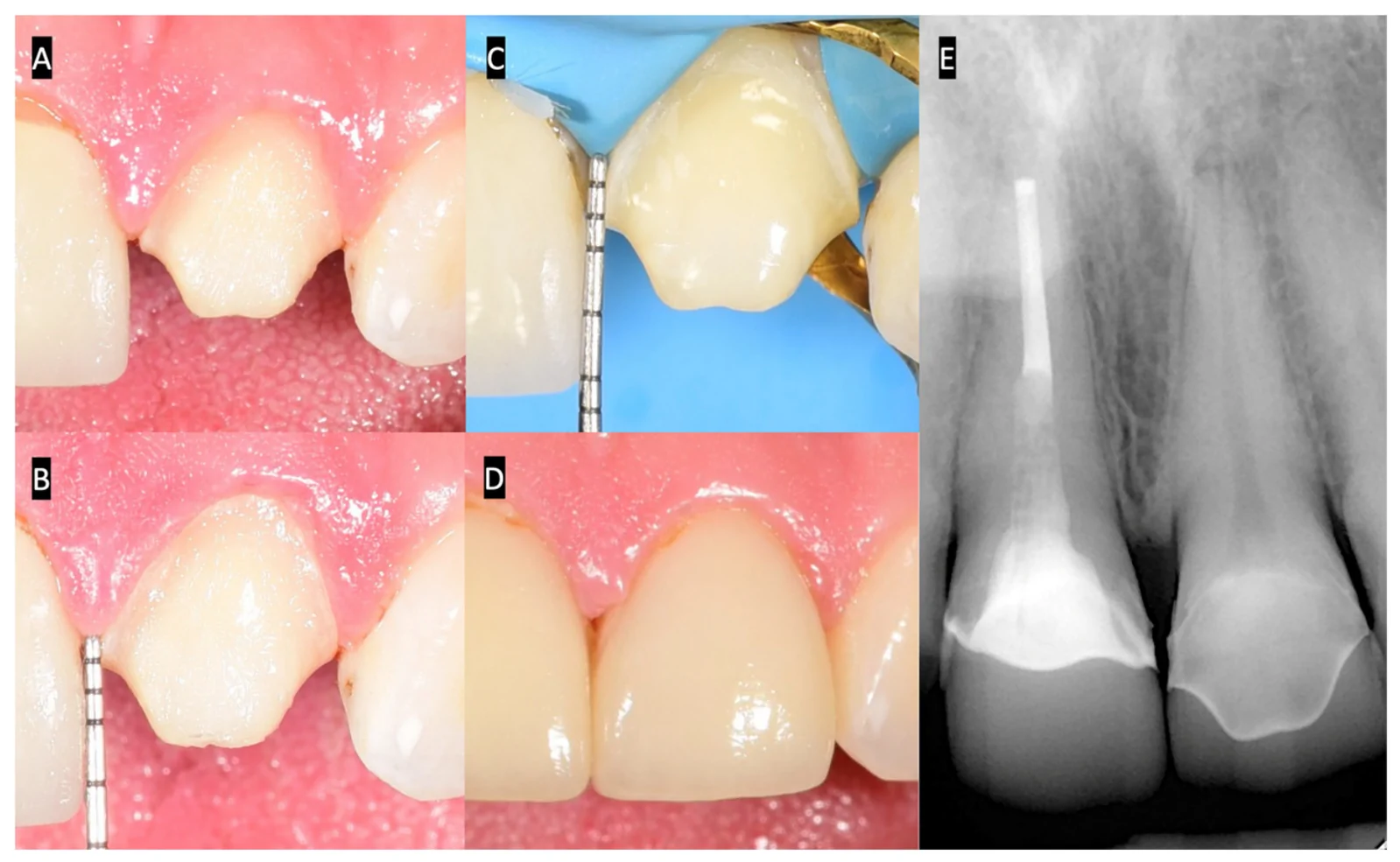
Zone 1 clinical scenario. (**A**) Initial clinical situation; (**B**) evaluation of margin depth with a periodontal probe; (**C**) treatment under dental dam isolation, showing the new margin depth evaluated with a periodontal probe; (**D**) final restoration; and (**E**) final radiograph after crown cementation.

This zone includes margins that are located at, or just slightly subgingival, without extending into the junctional epithelium. In most cases, these margins can be managed with conventional rubber dam isolation, which provides enough soft tissue displacement to ensure good visibility and moisture control. As a result, procedures in this zone are generally more predictable and less technique-sensitive. Standard matrix systems are typically sufficient to achieve proper contour and marginal adaptation (Figure 2).

### 2.3. Zone 2—Margins Within the Junctional Epithelium and Supracrestal Tissue Attachment

**Figure 3:**
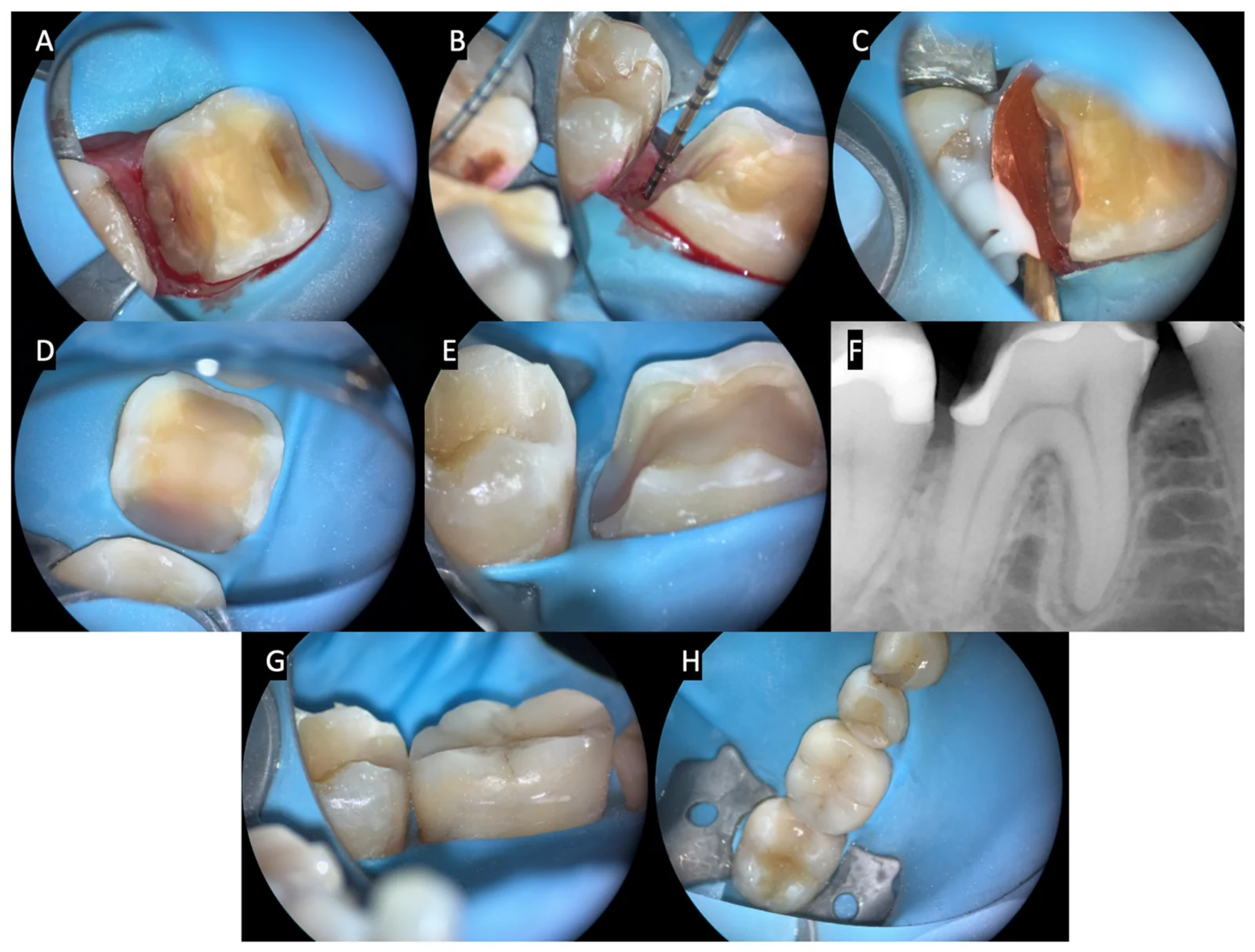
Zone 2 clinical scenario. (**A**) Initial occlusal view; (**B**) initial lateral view with evaluation of margin depth using a periodontal probe; (**C**) occlusal view after placement of the matrix; (**D**) occlusal view after completion of deep margin elevation; (**E**) lateral view after completion of deep margin elevation; (**F**) radiograph of the deep margin elevation; (**G**) lingual view of the final full-coverage crown; and (**H**) occlusal view of the final full-coverage crown.

Margins in this zone extend into the junctional epithelium and supracrestal connective tissue attachment. Because these two components cannot always be clearly distinguished clinically, they are considered together. Management is more challenging because access, hemostasis, isolation, and matrix adaptation may be compromised. Sectional or circumferential matrices, wedges, and soft tissue displacement may help improve access and stabilization. Adhesive procedures at this level are highly technique-sensitive and require careful control of blood, crevicular fluid, and saliva. In selected cases, DME may be feasible; however, DME alone may not always be sufficient. When adequate isolation, access, or a biologically acceptable margin cannot be achieved, adjunctive surgical procedures, such as crown lengthening, may need to be considered. Adhesive procedures in this area are more technique-sensitive and depend heavily on careful control of the working field (Figure 3).

### 2.4. Zone 3—Margins at or Below the Alveolar Bone Crest (Intraosseous Level)

**Figure 4:**
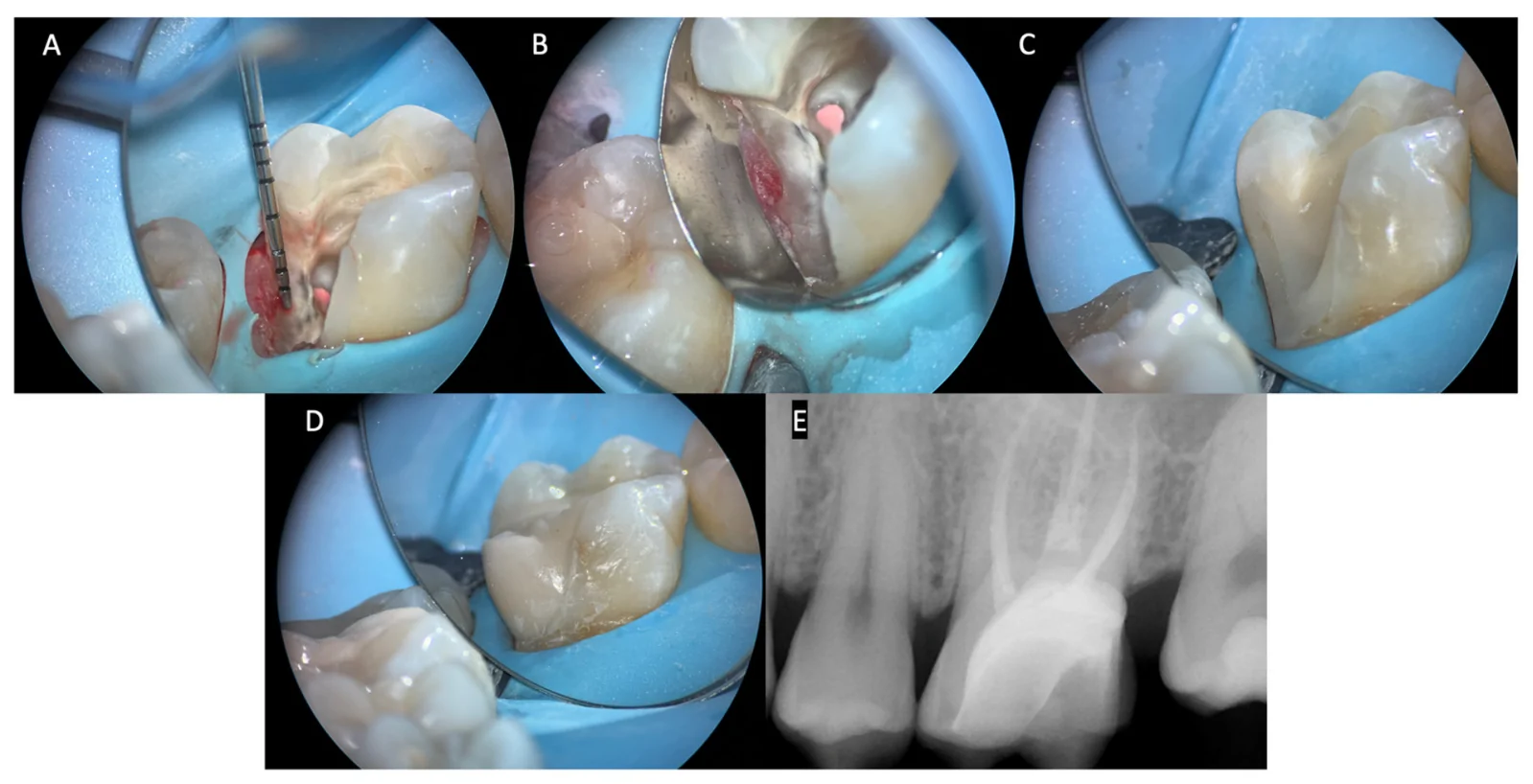
Zone 3 clinical scenario. (**A**) Initial lateral view with evaluation of margin depth using a periodontal probe; (**B**) lateral view of matrix placement; (**C**) completion of deep margin elevation; (**D**) final restoration; and (**E**) final radiograph.

This zone includes margins located at or below the level of the alveolar bone crest, where the supracrestal tissue attachment is compromised. Zone 3 should be considered a high-risk clinical scenario rather than a routine indication for DME. In many cases, surgical management—most commonly crown lengthening—may be required to re-establish a biologically acceptable relationship between the restorative margin and the periodontal tissues while also improving access for treatment. In carefully selected cases, advanced restorative approaches such as double-matrix or matrix-in-matrix techniques, using rigid materials such as stainless steel matrices or copper bands, may help relocate the margin coronally. However, these procedures are highly technique-sensitive, carry greater biological risk, and should be considered only after thorough case assessment and when adequate isolation, access, and periodontal conditions can be achieved (Figure 4).

## 3. Results

The proposed classification has practical relevance, particularly when it comes to isolation, which is essential for the success of adhesive procedures such as deep margin elevation (DME). In Zone 1, isolation is usually straightforward, and a conventional rubber dam is often sufficient to provide adequate moisture control and slight displacement of the surrounding soft tissues. In contrast, managing margins in Zones 2 and 3 is more demanding. In these areas, controlling sulcular fluid and bleeding becomes more challenging, and additional measures such as wedges, retraction techniques, and careful matrix placement are often required. As margins extend further apically, maintaining a clean and dry working field becomes increasingly difficult, which in turn makes adhesive procedures more technique-sensitive.

Matrix selection and adaptation should also be tailored to the anatomical zone. For Zone 1, standard sectional or circumferential matrices are generally adequate. In Zone 2, the deeper position of the margin requires improved adaptation, often achieved by combining matrices with wedges to ensure proper sealing and contour. In Zone 3, access is significantly restricted, and more advanced techniques may be needed. Approaches such as double-matrix or matrix-in-matrix techniques, often using rigid materials like stainless steel strips or copper bands, can help stabilize the matrix and improve marginal adaptation. However, the evidence supporting these methods is still limited.

This classification may also assist in choosing between restorative and surgical treatment approaches. Margins located in Zone 1 and, in some cases, Zone 2 can often be managed predictably with restorative techniques alone. When margins extend into Zone 3 and compromise the supracrestal tissue attachment, surgical intervention, such as crown lengthening, remains the most established option for restoring both periodontal health and adequate access. Restorative alternatives in these situations should be approached with caution, taking into account the potential biological consequences and the limited long-term data available. Table 1 summarizes the proposed classification system.

## 4. Discussion

The deep margin elevation procedure is a complex restorative approach because it often requires caries removal in subgingival areas. During the procedure, the clinician must control bleeding, crevicular fluid, and the surrounding soft tissues throughout both caries removal and restoration placement. The literature includes several reports describing different approaches to these clinical scenarios and their associated outcomes. A summary of the available evidence is presented in Table 2 [13,14,15,16,17].

The proposed classification is based on well-established biological principles related to the supracrestal tissue attachment, which governs the relationship between restorative margins and the surrounding periodontal tissues. Both classic and more recent studies have shown that disruption of this attachment can lead to inflammation, loss of attachment, and even bone resorption [18,19]. By dividing subgingival margins into three zones, this model aims to translate those biological concepts into something more clinically practical. Rather than focusing only on exact measurements, it takes into account how accessible a margin is and how it can realistically be managed during treatment, while also recognizing that periodontal anatomy can vary considerably from one case to another.

Zone 1 includes margins located at, or only slightly apical to, the gingival margin without extending into the junctional epithelium. This represents the least complex clinical scenario and may be more readily managed by less experienced clinicians. Because of the relatively superficial depth of the margin, a dental dam can generally be positioned to provide adequate access and complete isolation of the operative field. Conventional matrix systems can also be used without substantial modification. Case reports in the literature have described successful deep margin elevation (DME) procedures in situations involving margins at this relatively shallow depth, demonstrating that a predictable restorative outcome can be achieved while addressing the patient’s restorative needs [20,21].

Zone 2 represents a more complex clinical situation because the lesion extends into the junctional epithelium and supracrestal tissue attachment. At this depth, access and isolation become more challenging, and the dental dam may require modification to improve visibility and moisture control. Conventional matrix systems may still be used, although additional stabilization, tissue displacement, and wedge placement are often necessary to achieve adequate adaptation. Careful control of bleeding, gingival crevicular fluid, and saliva is essential because contamination may compromise the adhesive procedure. Although DME may be feasible in selected Zone 2 cases, the outcome is highly dependent on local anatomy, tissue conditions, and operator experience. In some situations, DME alone may not provide adequate access or a biologically acceptable margin position, and adjunctive surgical procedures, such as crown lengthening, may need to be considered [22,23].

Zone 3 represents the most challenging and highest-risk clinical scenario because the restorative margin is located at, or extends apical to, the level of the alveolar bone crest. This zone should not be considered a routine indication for DME. At this depth, conventional matrix systems may lack sufficient rigidity or stability, and advanced approaches such as double-matrix techniques, matrix-in-matrix techniques, copper bands, or rigid stainless-steel matrices may be required to improve adaptation and resist displacement from the surrounding tissues and bone. Rubber dam isolation may still be attempted, but significant modification or displacement is often necessary, and in some cases adequate isolation may not be achievable. Because of the proximity to the alveolar crest and the potential compromise of the supracrestal tissue attachment, management of Zone 3 lesions is highly technique-sensitive and biologically demanding. Meticulous control of bleeding, moisture, matrix adaptation, and restorative contour is essential. Although clinical reports have described successful management of margins at this depth, these situations require careful case selection and substantial operator experience. In many cases, DME alone may not provide a biologically acceptable or clinically manageable result, and surgical intervention, such as crown lengthening, should be considered. Therefore, Zone 3 should be regarded primarily as a high-risk scenario requiring comprehensive evaluation, rather than as a predictable or routine candidate for DME [24,25].

It is also important to recognize that other factors, such as root morphology, gingival biotype, defect configuration, and tooth position, are critical when assessing the feasibility of performing DME. In some clinical situations, a thorough initial evaluation may indicate that additional procedures, such as crown lengthening or bone augmentation, are necessary. Therefore, careful assessment of these factors is essential before initiating treatment.

When considering surgical management, crown lengthening is still the most predictable and widely accepted approach for margins that extend into or beyond the supracrestal tissue attachment. It allows for the re-establishment of a healthy periodontal environment and provides better access for restorative procedures. At the same time, it is not without drawbacks, including the removal of supporting bone, possible esthetic compromises, and a longer treatment process [26,27,28,29,30]. The aim of this classification is not to replace surgical treatment, but to help clarify in which situations restorative approaches such as deep margin elevation may be appropriate, and when surgery remains the more biologically sound option.

Several limitations should be considered when interpreting the proposed classification. First, it is conceptual and has not yet been prospectively validated. The three zones are based on periodontal biological principles, anatomical relationships, and anticipated clinical difficulty rather than on universally accepted quantitative thresholds. In addition, the dimensions of the supracrestal tissue attachment vary among patients, teeth, and tooth surfaces, and can be influenced by factors such as gingival phenotype, periodontal health, tooth morphology, and alveolar crest position. These variations, together with the limitations of periodontal probing and two-dimensional radiographs, may affect the consistency and reproducibility of assigning a lesion to a specific zone.

A second limitation is that the classification is primarily based on the apicocoronal depth of the lesion, even though clinical complexity is influenced by several additional factors. The buccolingual and mesiodistal extension of the lesion, circumferential involvement, tooth position, root anatomy, remaining tooth structure, pulpal and endodontic status, occlusal loading, and accessibility may all affect treatment difficulty and prognosis. Therefore, lesions within the same zone may present different levels of complexity. In addition, outcomes may be influenced by the restorative material, matrix system, adhesive protocol, isolation technique, and the clinician’s experience, particularly in deeper lesions where hemostasis, tissue management, and matrix adaptation become increasingly technique-sensitive.

Finally, the proposed classification should not be interpreted as an absolute indication or contraindication for deep margin elevation, particularly in Zone 3 lesions that approach or extend to the alveolar crest. In such cases, alternative treatments such as surgical crown lengthening, orthodontic extrusion, or extraction may need to be considered based on the overall biological and restorative prognosis. The classification also does not yet account for long-term periodontal changes, including gingival recession, attachment loss, inflammation, or bone remodeling. Consequently, further clinical studies are needed to determine its reproducibility, predictive value, and association with restorative and periodontal outcomes. Until such evidence is available, the classification should be considered a practical framework for education, communication, and clinical decision-making rather than a definitive treatment protocol.

## 5. Conclusions

The three-zone classification proposed in this paper offers a practical, anatomy-based way of assessing subgingival margins. By relating the position of the margin to both periodontal structures and clinical accessibility, it may help clinicians choose appropriate isolation methods, matrix systems, and overall treatment strategies in procedures such as deep margin elevation. In this way, it has the potential to support more consistent and better-informed clinical decision-making.

That said, the classification is still conceptual and has not yet been clinically validated. Further research is needed to evaluate how reliably it can be applied in practice, as well as its influence on both periodontal health and restorative outcomes. Well-designed clinical studies will be essential before it can be considered for broader adoption as a standard guideline.

## Figures and Tables

**Table 1:** Description of the three zone’s classification.

Zone	Anatomical Location	Clinical Complexity	Isolation/Matrix Considerations	Clinical Considerations
Zone 1	Margin at or slightly apical to the gingival margin, without extension into the junctional epithelium	Low	Conventional dental dam isolation and standard matrix systems are usually adequate	Generally predictable and less technique sensitive
Zone 2	Margin extends into the junctional epithelium and supracrestal tissue attachment	Moderate	Dental dam modification, edges, tissue displacement, and sectional or circumferential matrices may be required	Increased risk of bleeding and moisture contamination; careful hemostasis and matrix adaptation are essential.
Zone 3	Margin located at or apical to the alveolar bone crest	High	Conventional matrices may be insufficient, rigid matrices, double-matrix technique, stainless-steel matrices, or copper bands may be required.	Highly technique-sensitive, isolation may be difficult, and surgical approaches such as crown lengthening may need to be considered.

**Table 2:** Summary of the clinical evidence available about deep margin elevation technique.

Title, Authors and Year of Publication	Methodology	Conclusion
Deep margin elevation and lithium disilicate overlay restoration of an endodontically treated molar: A case report. Yousfi et al., 2026 [13]	A 38-year-old patient presented with recurrent debonding of a partial indirect restoration on tooth 47, which had previously undergone endodontic treatment. A deep occlusoproximal defect with a subgingival cervical margin was identified. Deep margin elevation (DME) was performed using a bulk-fill composite resin to relocate the margin to a supragingival position.	The procedure appears to provide a conservative treatment approach that preserves dental tissues and periodontal health, supporting the potential clinical applicability of this technique.
Deep margin elevation in anterior teeth: A clinical report. Grassi et al., 2025 [14]	A 19-year-old woman presented with trauma to the maxillary anterior region following a bicycle accident. The fracture of the maxillary right central incisor extended subgingivally. DME was performed on the palatal surface, followed by placement of a definitive ceramic veneer.	The 6-year clinical follow-up demonstrated that DME can be effective for managing deep margins by improving visibility and facilitating a dry working field for partial ceramic restorations in the anterior region.
Elevating subgingival margins: A case series in deep margin management. Arora et al., 2024 [15]	Case 1: A 67-year-old man presented with deep distal caries on the mandibular left first molar. Treatment included DME followed by a Class II composite restoration. Case 2: A 24-year-old man presented with deep proximal caries on the maxillary right first molar. Treatment included DME followed by a full-coverage crown.	DME represents a conservative and reliable approach for managing subgingival defects, provided that the procedure is performed carefully to achieve a smooth, well-sealed restorative surface that supports proper adaptation and periodontal health.
Deep margin elevation for indirect bonded restorations: A case report. Ayari et al., 2021 [16]	A 30-year-old woman presented with extensive secondary caries extending below the cementoenamel junction. The lesion was treated using DME followed by a Class II restoration.	DME is a relatively recent technique with recognized clinical benefits. The longevity of indirect restorations depends on adherence to an appropriate clinical protocol and the use of suitable restorative materials.
Deep margin elevation with one-year follow-up: A case report. Aljanakh, 2024 [17]	A 57-year-old woman presented with deep distal caries on the maxillary left first molar. Treatment included DME of the distal margin followed by placement of a full-coverage crown.	This case report suggests that DME can serve as a conservative treatment strategy consistent with contemporary evidence-based dental practice when appropriate patient selection and clinical protocols are followed.

## Data Availability

The original contributions presented in the study are included in the article; further inquiries can be directed to the corresponding author.
